# Upregulated EMMPRIN/CD147 might contribute to growth and angiogenesis of gastric carcinoma: a good marker for local invasion and prognosis

**DOI:** 10.1038/sj.bjc.6603425

**Published:** 2006-10-31

**Authors:** H-C Zheng, H Takahashi, Y Murai, Z-G Cui, K Nomoto, S Miwa, K Tsuneyama, Y Takano

**Affiliations:** 1 Department of Diagnostic Pathology, Graduate School of Medical and Pharmaceutical Sciences, University of Toyama, Toyama, Japan; 2 21st Century COE Program, Graduate School of Medical and Pharmaceutical Sciences, University of Toyama, Toyama, Japan; 3 Department of Internal Medicine (3), Graduate School of Medical and Pharmaceutical Sciences, University of Toyama, Toyama, Japan

**Keywords:** gastric carcinoma, progression, angiogenesis, prognosis, EMMPRIN

## Abstract

Tumour growth depends on angiogenesis, which is closely associated with vascular endothelial growth factor (VEGF) and matrix metalloproteinases (MMPs). Extracellular MMP inducer (EMMPRIN) was reported to involve in the progression of malignancies by regulating expression of VEGF and MMPs in stromal cells. To clarify the role of EMMPRIN in progression and angiogenesis of gastric carcinoma, expression of EMMPRIN, ki-67, MMP-2, MMP-9 and VEGF was examined on tissue microarray containing gastric carcinomas (*n*=234) and non-cancerous mucosa adjacent to carcinoma (*n*=85) by immunohistochemistry. Additionally, microvessel density (MVD) was assessed after labelling with anti-CD34 antibody. Extracellular MMP inducer expression was compared with clinicopathological parameters of tumours, including levels of ki-67, MMP-2, MMP-9 and vascular endothelial growth factor (VEGF), MVD as well as survival time of carcinoma patients. Gastric carcinoma cell lines (HGC-27, MKN28 and MKN45) were studied for EMMPRIN expression by immunohistochemistry and Western blot. Extracellular MMP inducer expression was gradually increased from normal mucosa to carcinomas through hyperplastic or metaplastic mucosa of the stomach (*P*<0.05). There was strong EMMPRIN expression in all gastric carcinoma cell lines despite different levels of glycosylation. Extracellular MMP inducer expression was positively correlated with tumour size, depth of invasion, lymphatic invasion, expression of ki-67, MMP-2, MMP-9 and VEGF of tumours (*P*<0.05), but not with lymph node metastasis, UICC staging or differentiation (*P>*0.05). Interestingly, there was a significantly positive relationship between EMMPRIN expression and MVD in gastric carcinomas (*P*<0.05). Survival analysis indicated EMMPRIN expression to be negatively linked to favourable prognosis (*P*<0.05), but not be independent factor for prognosis (*P*>0.05). Further analysis showed three independent prognostic factors, depth of invasion, lymphatic and venous invasion, to influence the relationship between EMMPRIN expression and prognosis. Upregulated expression of EMMPRIN possibly contributes to genesis, growth and local invasion of gastric carcinomas. Altered EMMPRIN expression might enhance growth, invasion and angiogenesis of gastric carcinoma via upregulating MMP expression of both stromal fibroblasts and gastric cancer cells and could be considered as an objective and effective marker to predict invasion and prognosis.

Growth of solid tumours depends on angiogenesis, a complex and multistep process which facilitates metastasis formation by increasing the likelihood of tumour cells to enter the blood circulation, provides nutrients and oxygen for growth at the metastasis site (Rundhaug, 2005). Critical steps during tumour angiogenesis are the outgrowth of endothelial cells from pre-existing capillary vessel and their migration from parental vessels under the stimulation of vascular endothelial growth factor (VEGF). Proliferating endothelial cells subsequently remodel the extracellular matrix (ECM) via matrix metalloproteinases (MMPs), align into tube-like structures, and eventually form new functional blood vessels. In cancer cells, overexpression of angiogenetic factors (eg, VEGF, MMPs, etc.) results in their more secretion into the ECM to stimulate the proliferation and mobility of vascular epithelial cells, closely linked to invasion and metastasis of malignancies (Rundhaug, 2005; Ueda *et al*, 2005).

The search for MMP-inducing factors in tumour cells leads to the identification of extracellular MMP inducer (EMMPRIN), whose name reflects its EMMPRIN activity. Structurally, EMMPRIN is a highly glycosylated (HG) cell surface transmembrane protein which belongs to the immunoglobulin superfamily, and is composed of two immunoglobulin domains in the extracellular region, a single transmembrane domain and a short cytoplasmic domain containing 39 amino acids (Muramatsu and Miyauchi, 2003; Gabison *et al*, 2005a, 2005b). Cotransfection of EMMPRIN expression vectors with different tags and crosslinking experiments have suggested that the molecules could associate with each other on the plasma membrane, forming homo-oligomers in a *cis*-dependent manner via the N-terminally located Ig-like domain (Yoshida *et al*, 2000; Sun and Hemler, 2001). Although EMMPRIN has a broader tissue distribution and is expressed on activated T cells, on differentiated macrophage, on retinal pigment epithelium, in the endometrium and in normal human keratinocytes, its expression is often elevated on breast cancers, hepatomas, oesophageal and cervical squamous cell carcinomas, colorectal and ovarian carcinomas, which increases tumour invasion by inducing MMP synthesis of surrounding stromal cells, including membrane type 1 and type 2 MMP, and the endogenous activators of MMP-2 (Davidson *et al*, 2003; Ishibashi *et al*, 2004; Marieb *et al*, 2004; Gabison *et al*, 2005a, 2005b; Li *et al*, 2005; Tang *et al*, 2005; Zhou *et al*, 2005; Sier *et al*, 2006; van der Jagt *et al*, 2006). Recently, various *in vivo* evidences indicated that EMMPRIN might stimulate tumour angiogenesis by elevating VEGF and MMP expression in the neighbouring fibroblasts and epithelial cells in a paracrine manner (Tang *et al*, 2005). Taken together, it was believed that EMMPRIN had strong impact on the invasion and metastasis of malignant tumours.

Gastric carcinoma ranks the world's second leading cause of cancer mortality behind lung cancer despite a sharp worldwide decline in both its incidence and mortality since the second half of the 20th century (Kelley and Duggan, 2003). Tumorigenesis and progression of gastric carcinoma is a multistage process with the involvement of a multifactorial aetiology, which mainly results from gene–environment interactions. In our study, EMMPRIN expression was for the first time examined in gastric carcinoma and non-cancerous mucosa adjacent to carcinoma (NCMAC), and compared with its clinicopathological parameters of tumours, including expression of ki-67, MMP-2, MMP-9 and VEGF proteins as well as prognosis to explore the roles of EMMPRIN in stepwise development of gastric carcinoma.

## MATERIALS AND METHODS

### Subjects

Gastric carcinomas (*n*=234) and NCMACs (*n*=85, 38 cases of normal mucosa and 47 cases of hyperplastic or metaplastic mucosa) were collected from our affiliated hospital and related institutes between 1993 and 2002. The patients with gastric carcinoma were 170 men and 64 women (38–88 years, mean=66.8 years). Among them, 104 cases have tumours accompanied with lymph node metastasis. None of the patients underwent chemotherapy or radiotherapy before surgery. They all provided consent for use of tumour tissue for clinical research and our University Ethical Committee approved the research protocol. We followed up carcinoma patients by consulting their case documents and telephoning.

### Pathology

All tissues were fixed in 4% neutralised formaldehyde, embedded in paraffin and incised into 4 *μ*m sections. These sections were stained by haematoxylin and eosin (HE) to confirm their histological diagnosis and other microscopic characteristics. The staging for each gastric carcinoma was evaluated according to the Union Internationale Contre le Cancer (UICC) system for the extent of tumour spread (Sobin and Wittekind, 2002). Furthermore, tumour size, depth of invasion, lymphatic and venous invasion, and differentiation were determined.

### Tissue microarray

**Figure 1:**
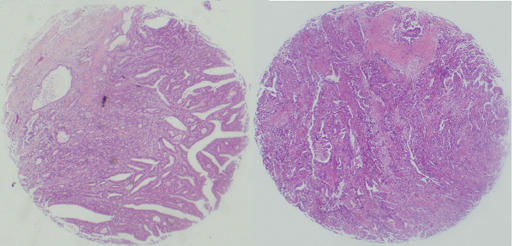
Haematoxylin and eosin staining on the tissue microarray of gastric carcinoma.

Representative areas of solid tumours were identified in HE-stained sections of the selected tumour cases and a 2 mm-in-diameter tissue core per donor block was punched out and transferred to a recipient block with a maximum of 48 cores using a Tissue Microarrayer (AZUMAYA KIN-1, Japan). Four-*μ*m-thick sections were consecutively incised from the recipient block and transferred to poly-lysine-coated glass slides. Haematoxylin and eosin staining was performed on tissue microarray for confirmation of tumour tissue (Figure 1).

### Cell lines and culture

Gastric carcinoma cell lines, HGC-27 (undifferentiated adenocarcinoma), MKN28 (well-differentiated adenocarcinoma) and MNK45 (poorly differentiated adenocarcinoma), come from Japanese Physical and Chemical Institute. They were maintained in RPMI 1640 (MKN28 and MKN45) or MEM (HGC-27) medium supplemented with 10% foetal bovine serum, 100 U ml^−1^ penicillin and 100 *μ*g ml^−1^ streptomycin, in a humidified atmosphere of 5% CO_2_ at 37°C. Total protein was prepared from all cells by cell disruption buffer according to PARIS manual (Ambion Inc, Austin, Texas, USA). All cells were collected by centrifugation, rinsed with phosphate-buffered saline, fixed by 10% formalin and then embedded in paraffin.

### Immunohistochemistry

Consecutive sections were deparaffinised with xylene, dehydrated with alcohol and subjected to antigen retrieval by irradiating in target retrieval solution (TRS, Dako, Carpinteria, CA 93013, USA) for 5 min with microwave oven (Oriental rotor Lmt. Co. Japan). Five per cent bovine serum albumin was then applied for 1 min to prevent nonspecific binding. The sections were incubated with primary antibodies for 15 min, then treated with the anti-mouse or anti-rabbit Envison-PO (Dako, USA) antibodies for 15 min. All the incubations were performed in a microwave oven to allow intermittent irradiation as described previously (Kumada *et al*, 2004). After each treatment, the slides were washed with TBST (10 mM Tris-HCl, 150 mM NaCl, 0.1% Tween 20) three times for 1 min. Mouse anti-EMMPRIN (Novocastro, New Castle upon Tyne NE 1282W, UK; 1 : 50), rabbit anti-ki-67 (Dako, USA; 1 : 25), mouse anti-MMP-2 (Daiichi Fine Chemical. Co. Lt, Japan; 1 : 50), mouse anti-MMP-9 (Daiichi Fine Chemical. Co. Lt, Japan; 1 : 150), rabbit anti-VEGF (LAB VISION, Fremont CA 94530, USA; ready to use), and mouse anti-CD34 (Dako, USA; 1 : 100) antibodies were employed for the detection of the respective proteins. Binding sites were visualised with 3,3′-diaminobenzidine. After counterstained with Mayer's haematoxylin, the sections were dehydrated, cleared and mounted. Omission of the primary antibody was used as a negative control and appropriate positive controls were utilised as recommended by the manufacturers.

### Evaluation of immunohistochemistry

**Figure 2:**
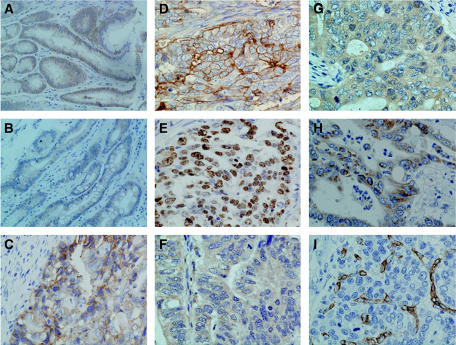
Immunostaining in gastric carcinoma and non-cancerous mucosa. Note EMMPRIN expression limited to cytoplasm and membrane in hyperplastic (**A**) and metaplastic (**B**) NCMAC and carcinoma (**C**, **D**) of the stomach. Ki-67 (**E**) distributed to the nucleus of gastric carcinoma cells, MMP-2 (**F**), MMP-9 (**G**) and VEGF (**H**) to the cytoplasm, and CD34 (**I**) in the membrane and cytoplasm of vascular epithelial cell to label MVD.

The immunoreactivity to EMMPRIN was localised in the cytoplasm and membrane, ki-67 in the nucleus, and MMP-2, MMP-9 and VEGF only in the cytoplasm (Figure 2). One hundred cells were randomly selected and counted from five representative fields of each section blindly by three independent observers (Takano Y, Tsuneyama K and Zheng HC). The positive percentage of counted cells was graded semiquantitatively according to a four-tier scoring system: negative (−), 0∼5%; weakly positive (+), 6∼25%; moderately positive (++), 26∼50% and strongly positive (+++), 51∼100%.

### Microvessel density counting

CD34 expression in the cytoplasm and membrane of vascular epithelial cells was selected for the microvessel density (MVD) counting, although it was occasionally localised in tumour cells and fibroblasts. A modified Weidner's method was used to calculate the MVD of gastric carcinoma after anti-CD34 immunostaining (Weidner, 1995). In brief, observers selected five representative areas and counted individual microvessels on a × 400 field (0.1885 mm^2^ per field) after the area of highest neovascularisation was identified, Any brown staining endothelial cell or endothelial cell cluster that was clearly separated from the adjacent microvessel, tumour cells and other connective tissue elements was considered as a single, countable microvessel. The counts were performed independently by two investigators.

### Western blot

Fifty micrograms of denatured protein was separated on an SDS–polycrylamide gel (10% acrylamide) and transferred to Hybond membrane (Amersham, Germany), which was then blocked overnight in 5% milk in TBST. For immunobloting, the membrane was incubated for 1 h with the antibody against EMMPRIN (Novocastra, UK, 1 : 100). Then, it was rinsed by TTBS and incubated with anti-mouse IgG conjugated to horseradish peroxidase (Dako, USA, 1 : 1000) for 1 h. Bands were visualised with X-ray film (Fujifilm, Japan) by Amersham ECL-Plus detection reagents (Amersham, Germany). After that, membrane was washed with WB Stripping Solution (pH2-3, Nacalai, Japan) for 1 h and treated as described above, except mouse tubulin-*α* antibody (Dako, USA, 1 : 100) as internal control.

### Statistical analysis

Statistical evaluation was performed using *Spearman* correlation test to analyse the rank data and *Kruskal–wallis H* test to differentiate non-parametric means of different groups. *Kaplan–Meier* survival plots were generated and comparisons between survival curves were made with the log-rank statistic. The Cox's proportional hazard model was employed for multivariate analysis. *P*<0.05 was considered as statistically significant. SPSS 10.0 software was employed to analyse all data.

## RESULTS

### Extracellular MMP inducer expression in gastric carcinomas

Extracellular MMP inducer expression was evident in the cytoplasm and membrane of hyperplastic, metaplastic epithelial cells of NCMAC and cancerous cells of stomach (Figure 2A, B). As showed in Table 1, EMMPRIN expression was increased from gastric normal mucosa to carcinoma through hyperplastic or metaplastic mucosa (*P*<0.05). As summarised in Table 2, EMMPRIN expression showed a significantly positive association with tumour size, depth of invasion, lymphatic invasion, expression of ki-67, MMP-2, MMP-9 and VEGF of tumours (*P*<0.05), but not with lymph node metastasis, UICC staging or differentiation (*P>*0.05). Interestingly, there was a significantly positive relationship between EMMPRIN expression and MVD in the gastric carcinomas (*P*<0.05) (Table 3).

**Figure 3:**
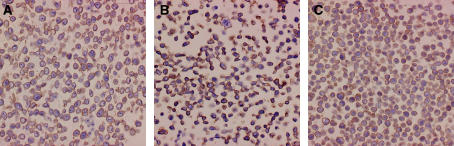
Extracellular MMP inducer immunostaining in gastric carcinoma cell lines. (**A**) HGC-27; (**B**) MKN28; (**C**) MKN45.

**Figure 4:**
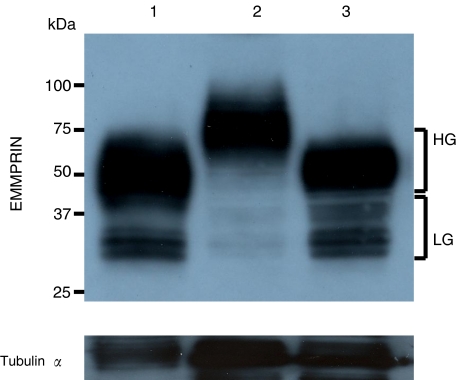
Western blot analysis of EMMPRIN in gastric carcinoma cell lines. Cell lysate was loaded and probed with monoclonal mouse anti-human EMMPRIN antibody with tubulin-*α* as an internal control. Lane no. 1: HGC-27; no. 2 MKN28; no. 3 MKN45. HG: highly glycosylated form (∼45–65 kDa); LG: less glycosylated form (∼32–44 kDa).

Three gastric cell lines exhibited strong EMMPRIN expression according to immunostaining data (Figure 3). Extracellular MMP inducer exists in tumour and transformed cell lines as HG form migrating at ∼45–65 kDa and as a less glycosylated (LG) form migrating at ∼32–44 kDa, depending on the glycosylation of the core protein (27 kDa) (Riethdorf *et al*, 2006; Vigneswaran *et al*, 2006). Western blot also indicated these cell lines strongly expressed EMMPRIN, but the HG form in all cell lines and LG one strongly in HGC-27 and MKN45 and weakly in MKN28 (Figure 4).

### Univariate and multivariate survival analysis

**Figure 5:**
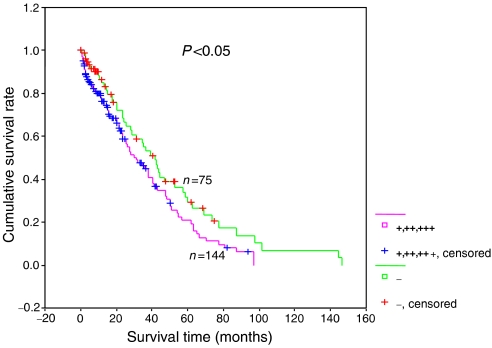
Correlation between status of EMMRIN and prognosis of the gastric carcinoma patients. Kaplan–Meier curves for cumulative survival rate of patients with gastric carcinomas according to EMMPRIN expression.

Follow-up information was available on 219 patients with gastric carcinoma for period ranging from 0.2 months to 12.2 years (mean=40.4 months). Figure 5 showed the survival curves stratified according to EMMPRIN expression. Univariate analyses using *Kaplan–Meier* method indicated that cumulative rate of the patients with negative EMMPRIN expression was significantly higher than that with its weakly, moderately and strongly positive expression (*P*<0.05). Multivariate analysis using the Cox's proportional hazard model indicated that depth of invasion, lymphatic and venous invasion, but not EMMPRIN, were independent prognostic factors (Table 4). Further analysis showed that these three locally invasive factors to have effects on the relationship between EMMPRIN expression and survival time of the carcinoma patients (Table 5).

## DISCUSSION

The microenvironment of the tumour–host interface is involved in many processes impacting on tumour development, including angiogenesis, growth, dissemination and metastasis of tumours. The crosstalk interaction between tumour cells and adjacent stromal cells participates in tumour immune escape, spreading and angiogenesis, which is conducted by a number of soluble and membrane molecules, including soluble Fas, Fas ligand, soluble MMPs, soluble VEGF and EMMPRIN (Zheng *et al*, 2003; Tang *et al*, 2005).

Numerous studies have indicated that the presence and modulation of EMMPRIN might play some role in the normal physiological processes (Gabison *et al*, 2005a, 2005b). In the present study, it was found that EMMPRIN was positively expressed in hyperplastic or metaplastic epithelium of gastric NCMAC. Gabison *et al* (2005a, 2005b) also reported EMMPRIN to be predominantly expressed in corneal epithelium but markedly elevated in the anterior stroma of ulcerated corneas. Therefore, we speculate that EMMPRIN might be involved in stromal remodelling and epithelial repair after injury. Compared with gastric normal, hyperplastic or metaplastic mucosa, gastric carcinoma highly expressed EMMPRIN protein in line with other malignancies (Davidson *et al*, 2003; Ishibashi *et al*, 2004; Marieb *et al*, 2004; Li *et al*, 2005; Tang *et al*, 2005; Zhou *et al*, 2005; Sier *et al*, 2006; van der Jagt *et al*, 2006). Additionally, strong expression was also observed in gastric carcinoma cell lines in our study. Experimental overexpression of EMMPRIN in relatively less aggressive carcinoma cells lines results in an ability to form large and malignant tumours with a more invasive phenotype in the nude mice (Toole, 2003). These findings suggest that upregulation of EMMPRIN plays an essential role in the malignant transformation of gastric epithelial cells.

Elevated EMMPRIN expression has also been shown to correlate with the progression of various malignancies (Davidson *et al*, 2003; Ishibashi *et al*, 2004; Marieb *et al*, 2004; Gabison *et al*, 2005a, 2005b; Li *et al*, 2005; Tang *et al*, 2005; Zhou *et al*, 2005; Sier *et al*, 2006; van der Jagt *et al*, 2006). Our results indicated that EMMPRIN overexpression was positively linked to tumour size of gastric carcinoma. To explore the mechanism about the regulatory effect of EMMPIN on tumour growth, we examined expression of ki-67, a good proliferation marker and consequently found a positive association between the two parameters. Yang *et al* (2006) found that EMMPRIN expression in breast carcinoma cells rendered them resistant to anoikis, a form of apoptosis triggered by a lack of or improper cell-matrix interactions, mediated by downregulation of the proapoptotic BH3-only protein, Bim, through an MAP kinase-dependent pathway. Marieb *et al* (2004) documented that upregulated EMMPRIN expression stimulates hyaluronan production by elevating hyoluronan synthases, which is closely related to the anchorage-independent growth of cancer cells. Taken together, our result supported the opinion that EMMPRIN might enhance tumour growth of gastric carcinomas by disrupting the balance between apoptosis and proliferation.

Our results showed no association between EMMPRIN expression and carcinoma differentiation, although its higher expression was found in intestinal-type gastric carcinoma (Zheng *et al*, 2006a, 2006b). The discrepancies might be largely attributable to different grouping and statistical methods. For example, the Spearman correlation analysis was employed to analyse the rank data in the present study, which could differentiate not only positive rate but also expression level. It was worth noting that LG EMMPRIN was weaker in the well-differentiated cell line (MKN28) than the poorly differentiated (MNK45) or undifferentiated ones (HGC-27), although there was no difference in its expression level from our statistical results of immunohistochemistry in gastric carcinoma, indicating that EMMPRIN glycosylation might be linked to carcinoma differentiation.

In our study, it was found that gastric carcinomas with higher EMMPRIN expression displayed more ability to invade into lymphatic or venous vessels, or through the gastric wall. A large body of evidence indicates that increased soluble EMMPRIN released from cancer cells can stimulate the MMP synthesis and activation of surrounding stromal cells so as to promote the progression of tumours (Yoshida *et al*, 2000; Sun and Hemler, 2001; Davidson *et al*, 2003; Muramatsu and Miyauchi, 2003; Ishibashi *et al*, 2004; Marieb *et al*, 2004; Gabison *et al*, 2005a, 2005b; Li *et al*, 2005; Tang *et al*, 2005; Zhou *et al*, 2005; Sier *et al*, 2006; van der Jagt *et al*, 2006). Recently, Tang *et al* (2004) indicated that elevation of MMPs mediated by EMMPRIN could result in more proteolytic cleavage of membrane-associated EMMPRIN, forming a positive feedback tumour-stoma interaction. Furthermore, EMMPRIN transfection of tumour cells or treating tumour cells with the recombinant protein increased the expression of MMPs, especially MMP-2 (Sun and Hemler, 2001), as also evidenced by the positive correlation of EMMPRIN expression with MMP-2 and MMP-9 expression in our cases of gastric carcinoma. Our group also found the negative association between expression of EMMPRIN and ECM tenascin, possibly owing to its regulatory effect on MMP secretion (Zheng *et al*, 2006a, 2006b). It was suggested that upregulated EMMPRIN expression possibly contributes to local invasion by stimulating the MMP expressions of both stromal and tumour cells in gastric carcinomas.

Tumour growth depends not only on apoptotic suppression, but also angiogenesis. In the present study, we found that there was a positive relationship between EMMPRIN expression and MVD in gastric carcinoma. A recent study indicated that EMMPRIN could enhance VEGF expression of stromal fibroblasts and carcinoma cells to stimulate tumour angiogenesis via the PI3K-Akt signalling pathway (Tang *et al*, 2005, 2006). Our results also showed that EMMPRIN expression was positively correlated with VEGF expression in gastric carcinoma. The EMMPRIN-induced MMP expression in cancer cells also had impact on the tumour angiogenesis (Sun and Hemler, 2001). In combination of these evidences, it was suggested that EMMPRIN-mediated angiogenesis might participate in growth and spread of gastric carcinoma.

Although Ishibashi *et al* (2004) reported that EMMPRIN expression was not associated with the recurrence-free survival of oesophageal squamous cell carcinoma, Davidson *et al* (2003) found that EMMPRIN was a good prognostic marker in ovarian carcinoma. To further clarify the clinicopathological significance, we analysed the relation of EMMPRIN expression with survival of 219 patients with gastric carcinoma. The results revealed a link between loss and favourable survival, albeit not independent of other parameters. The multivariate analysis demonstrated three independent prognostic factors, depth of invasion, lymphatic and venous invasion, which affected the relationship between EMMPRIN expression and prognosis.

In conclusion, upregulated expression of EMMPRIN might contribute to tumorigenesis, growth and local invasion of gastric carcinoma. Altered EMMPRIN expression might enhance invasion and angiogenesis via upregulating MMP and VEGF expression of both stromal fibroblasts or gastric carcinoma cells. It could thus be considered as an objective and effective marker to predict the invasion and prognosis of gastric carcinoma. The regulatory effects of EMMPRIN on VEGF in gastric carcinoma should be clarified in the further study.

## Figures and Tables

**Table 1:** EMMPRIN expression in gastric NCMAC and carcinoma

		**EMMPRIN expression**						
**Groups**	**n**	−	+	**++**	**+++**	**PR (%)**	***rs***	***P*-value**
Normal mucosa	38	26	10	2	0	31.6	0.288	<0.0001
Hyperplastic or metaplastic mucosa	47	17	20	9	1	63.8		
Carcinoma	234	82	51	47	54	65.0^*^		

EMMPRIN=extracellular matrix metalloproteinase inducer; NCMAC=non-cancerous mucosa adjacent to carcinoma; PR=positive rate; *rs*=Spearmen correlation coefficient.

**Table 2:** Relationship between EMMPRIN expression and clinicopathological features of gastric carcinoma

		**EMMPRIN expression**						
**Clinicopathological features**	**n**	−	+	**++**	**+++**	**PR (%)**	***rs***	***P-*value**
*Tumour size* (*cm*)							0.154	<0.05
<4 cm	110	44	27	21	18	60.0		
⩾4 cm	124	38	24	26	36	69.4		
								
*Depth of invasion*							0.142	<0.05
T_is_–T_1_	113	41	34	21	17	63.7		
T_2_–T_4_	121	41	17	26	37	66.1		
								
*Lymphatic invasion*							0.129	<0.05
−	140	51	39	24	26	63.6		
+	94	31	12	23	28	67.0		
								
*Venous invasion*							0.298	<0.001
−	206	78	49	41	37	62.1		
+	28	3	2	6	17	89.3		
								
*Lymph node metastasis*							0.094	>0.05
−	130	48	33	23	26	63.1		
+	104	34	18	24	28	67.3		
								
*UICC staging*							0.106	>0.05
O–I	141	52	36	25	28	63.1		
II–IV	93	30	15	22	26	67.7		
								
*Differentiation*							0.067	>0.05
Well	94	26	25	27	16	72.3		
Moderate	30	10	5	4	11	66.7		
Poor	110	46	21	16	27	58.2		
								
*Ki-67 expression*							0.184	<0.005
−	18	10	2	3	3	44.4		
+	34	14	7	7	6	58.8		
++	57	25	11	11	10	56.1		
+++	125	33	31	26	35	73.6		
								
*MMP-2 expression*							7.484	<0.001
−	21	13	5	2	1	38.1		
+	36	16	13	5	2	55.6		
++	72	30	18	12	12	58.3		
+++	90	16	12	26	36	82.2		
								
*MMP-9 expression*							0.378	<0.001
−	65	34	13	9	9	47.7		
+	31	15	8	5	3	51.6		
++	51	22	10	12	7	56.9		
+++	81	9	20	20	32	88.9		
								
*VEGF expression*							0.431	<0.001
−	47	31	12	2	2	34.0		
+	42	15	13	11	3	64.3		
++	37	15	7	9	6	59.5		
+++	103	21	19	24	39	79.6		

EMMPRIN=extracellular matrix metalloproteinase inducer; PR=positive rate; MMP=matrix metalloproteinase; *rs*: Spearmen correlation coefficient; T_is_=carcinoma *in situ*; T_1_=lamina propria and submucosa; T_2_=muscularis propria and subserosa; T_3_=exposure to serosa; T_4_=invasion into serosa; UICC=Union Internationale Contre le Cancer; VEGF=vascular endothelial growth factor.

**Table 3:** Relationship between EMMPRIN expression and MVD in gastric carcinoma

**EMMPRIN expression**	***n***	**MVD (mean±s.d.)**	***P*-value**
−	82	30.4±18.7	<0.05
+	50	28.6±12.6	
++	47	36.1±17.9	
+++	54	38.9±23.0	
			
Total	233	33.1±18.9	

EMMPRIN=extracellular matrix metalloproteinase inducer; MVD=microvessel density; s.d., standard deviation.

**Table 4:** Multivariate analysis of clinical variables for gastric carcinomas

**Number**	**Clinicopathological parameters**	**Relative risk (95% CI)**	***P*-value**
A	Tumour size (⩾4 cm)	1.496 (0.792–2.826)	>0.05
B	Depth of invasion (T_is,1_/T_2,3_)	3.635 (1.501–8.804)	<0.05
C	Lymphatic invasion (−/+)	1.576 (1.222–2.032)	<0.05
D	Venous invasion (−/+)	1.539 (1.068–2.218)	<0.05
E	Lymph node metastasis (−/+)	2.635 (0.913–7.607)	>0.05
F	UICC staging (O–I/II–IV)	0.429 (0.143–1.284)	>0.05
G	Differentiation (Well/moderately/poorly)	1.014 (0.778–1.322)	>0.05
H	Ki-67 expression (−/+/++/++++)	0.822 (0.619–1.092)	>0.05
I	MMP-2 expression (−/+/++/++++)	0.978 (0.706–1.357)	>0.05
J	MMP-9 expression (−/+/++/++++)	1.028 (0.836–1.264)	>0.05
K	VEGF expression (−/+/++/++++)	0.957 (0.748–1.225)	>0.05
L	MVD	1.001 (0.987–1.014)	>0.05
M	EMMPRIN expression (−/+∼++++)	1.055 (0.848–1.313)	>0.05

CI=confidence interval; EMMPRIN=extracellular matrix metalloproteinase inducer; MMP=matrix metalloproteinase; MVD=microvessel density; UICC=Union Internationale Contre le Cancer; VEGF=vascular endothelial growth factor.

**Table 5:** Multivariate analysis of EMMPRIN expression and other concordant factors in gastric carcinomas

**Groups**	**Relative risk (95%CI)**	***P-*value**
M	1.191 (0.917–1.415)	<0.05
M+B	1.085 (0.917–1.285)	>0.05
M+C	1.084 (0.912–1.290)	>0.05
M+D	1.042 (0.860–1.263)	>0.05
M+B+C	1.052 (0.887–1.248)	>0.05
M+B+D	0.990 (0.820–1.195)	>0.05
M+C+D	1.008 (0.837–1.214)	>0.05
M+B+C+D	0.982 (0.816–1.181)	>0.05

CI=confidence interval; EMMPRIN=extracellular matrix metalloproteinase inducer.
